# A Game System for Cognitive Rehabilitation

**DOI:** 10.1155/2015/493562

**Published:** 2015-03-01

**Authors:** Azrulhizam Shapi'i, Nor Azan Mat Zin, Ahmed Mohammed Elaklouk

**Affiliations:** Faculty of Information Science and Technology, Universiti Kebangsaan Malaysia (UKM), 43600 Bangi, Selangor, Malaysia

## Abstract

Brain injury such as traumatic brain injury (TBI) and stroke is the major cause of long-term disabilities in many countries. The increasing rate of brain damaged victims and the heterogeneity of impairments decrease rehabilitation effectiveness and competence resulting in higher cost of rehabilitation treatment. On the other hand, traditional rehabilitation exercises are boring, thus leading patients to neglect the prescribed exercises required for recovery. Therefore, we propose game-based approach to address these problems. This paper presents a rehabilitation gaming system (RGS) for cognitive rehabilitation. The RGS is developed based on a proposed conceptual framework which has also been presented in this paper.

## 1. Introduction

Brain injury such as traumatic brain injury (TBI) and stroke is the leading cause of long-term disabilities in many countries. Cognitive impairments occurring after sustaining a brain injury include attention, memory, and executive function deficiencies. These consequences dramatically affect patients' lives and limit the performance of their everyday activities [1].

There are many issues which are involved in decreasing rehabilitation effectiveness and competence. The increasing rate of brain damaged victims results in limited human resources and facilities, thus burdening the healthcare systems; for example, in the United States alone, 7% of the population or approximately 20 million people are suffering from cognitive disabilities [2]. In addition, the heterogeneity of impairments that patients suffer from is a relevant factor for planning, developing, and evaluating treatments. Hence, tailoring individualized rehabilitation tasks required for each patient involve a higher cost. The health care costs for patients after sustaining brain injuries are among the highest compared with other healthcare services in many countries [3].

On the other hand, studies revealed that majority of patients (75%) with traumatic brain injuries are less than 35 years in age [4], and this age group is more inclined to play games on computers and/or handheld games devices compared to other old age groups. Current research findings revealed that the brain has the ability to cure itself following an injury through repetitive, intensive, and task oriented training [5, 6]. However, brain damaged patients commonly reported that traditional rehabilitation exercises can be boring due to their repetitive nature which further lead them to neglect the exercises required for recovery [7]. In addition, patient's motivation is found to be an important factor for rehabilitation success and is often utilized as a determining factor in the outcome of rehabilitation [8]. The therapists' main problem is to find a way to encourage patients to actively take part in a rehabilitation program [9, 10]. Therefore, with such substantial effects on the quality of life of millions of patients and on healthcare systems worldwide, a feasible game-based intervention that can increase rehabilitation adequacy and effectiveness is crucial. This paper presents a rehabilitation gaming system (RGS) that would address these issues. The RGS is developed based on a proposed conceptual framework described in the following sections.

## 2. Proposed Conceptual Framework

**Figure 1:**
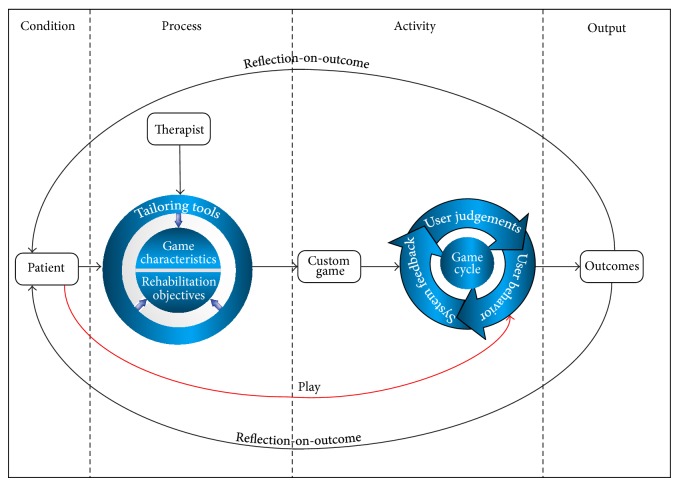
A conceptual framework for designing brain injury cognitive rehabilitation game.

Research on serious games for people with cognitive disabilities is still in its infancy, compared to other types of disabilities [11]. The proposed conceptual framework for designing a brain injury cognitive rehabilitation gaming system is illustrated in Figure 1. The aim is to establish a framework that can be used by the game developers and practitioners while designing game systems for cognitive rehabilitation. The framework was constructed from the results of our investigation and other related literatures [12–14]. Basically, the proposed framework consists of four components, namely, condition, process, activity, and outcome. Each one of these parts plays a crucial role in designing feasible game-based cognitive rehabilitation intervention that can increase rehabilitation effectiveness and competence. The following sections describe these components in detail.

### 2.1. Condition

The main factor that causes mismatch between a patient and potential technology is inadequate evaluation and assessment of the patient's needs and preferences [1, 15]. The difficulty of matching a patient and technology emerges not only from his unique combination of cognitive, sensory, and physical abilities but also from his expectations and reactions to the new technology interventions. These reactions arise from personal preferences, needs, abilities, and prior technology experiences [15].

Cognitive assessment practice typically begins with simple tests such as the Mini Mental State Exam (MMSE). In case of MMSE poor performance, it is fundamental to conduct a more in-depth evaluation using the neuropsychological assessment (NA) [16]. In addition, investigation of personal and health related factors as well as environment may help in the identification of factors that influence patient's functional ability and recovery [17]. The International Classification of Functioning, Disability and Health (ICF) model of human functioning and disability focuses on the “components of health” instead of the “consequences of disease” [18]. The ICF can be used as a valuable framework to manage and classify patient's information and it helps rehabilitation professionals in identifying aspects of a patient's condition that affect his/her recovery [19]. Therefore, game-based rehabilitation interventions are most effective when they are shaped to meet a patient's particular needs and preferences, not when they are prescribed as an isolated intervention of addressing particular cognitive and/or physical deficiencies. This process needs to begin with a comprehensive evaluation that gives the rehabilitation professionals an opportunity to assess the patient's ability, needs, preferences, and expectations. The evaluation generates information that can assist in tailoring game interventions which further enhance the quality of training and positively affect the self-esteem and motivation of patients.

### 2.2. Process

Garris et al. [20] described game characteristics in terms of six categories: fantasy, rules/goals, sensory stimuli, challenge, mystery, and control. They further argued that success in pairing game's characteristics with appropriate instructional practices could trigger individuals' motivational forces towards achieving the intended outcome. In our proposed framework as shown in Figure 1, the “tailoring tools” are responsible for mapping a game's characteristics with the intended rehabilitation objectives. For example, a “challenge” is a crucial game characteristic. Providing optimal challenge for a specific patient means matching the game difficulty to the patient's ability; being that it is neither too easy nor too difficult. To enable the optimal challenge, it is necessary to continuously adapt the game or create new game levels using “tailoring tools” to match the patient's existing skills.

Although a fully automated rehabilitation intervention which assesses the patient's deficiencies and then uses this assessment to create an individualized rehabilitation plan is virtually possible, such intervention has low chances to be medically accepted [21]. Rehabilitation professionals' experience in formulating and determining rehabilitation objectives and selecting exercises and facilities to attain those objectives has to be recognized. However, compromising between a fully automated rehabilitation intervention and a fully therapist-dependent rehabilitation intervention is probably the best alternative [21].

In a physical rehabilitation context, although some researchers have built games to address particular deficits, these customized games take time to create, are expensive, and cater only a small number of brain damaged patients. Environments with authoring tools that decrease time and expense enable therapists to quickly create games tailored to individuals with brain injury [22]. However, in cognitive rehabilitation, where there is diversity and heterogeneity of cognitive impairments, environments with “authoring tools” to create customizable games can be more cost-effective and can provide feasible games that can meet the specific needs of brain injured individuals.

The complexity of a game environment depends on its “tailoring tools” and ability of these tools to map the intended rehabilitation objectives with various game characteristics based on the needs and preferences of the patient. However, rehabilitation professionals often do not possess advanced knowledge and skills to understand the underlying design and development. Therefore, the game environment and its tailoring tools should be intuitive enough without the need of much technical knowledge.

### 2.3. Activity

A custom game is the output of the process part as shown in Figure 1, whereby the game is ready to be played by the patient. Retention of patient attention and his/her deep involvement depends on the effectiveness of the tailoring of these game activities by therapists. If the therapist succeeds in mapping the game's characteristics with the intended rehabilitation goal in the game, this will produce a repeating game cycle [20, 23]. The game cycle may help in sustaining patient's engagement in the rehabilitation intervention, which in turn leads to specific cognitive and affective outcomes.

### 2.4. Output

The game play activities generate specific outcomes which tell the level of patient achievement in playing the game. This achievement can be as simple as describing the game scores such as the total amount of assets collected and the time taken to achieve the goal within the game; or it can be extended to describe “changes of patient's outcomes,” which involves measuring improvements in a given cognitive function over time. This achievement can serve the purpose of patient assessment. Hence, new game playing activities should be modified and adapted to suit the patient's level. Therefore, outcomes play a crucial role and can be used as monitoring and tracking mechanisms by therapists. This promotes the possibility of unsupervised rehabilitation that can be continued after the patient is discharged from inpatient rehabilitation services. Thus, patients do not need long instructions and supervision by therapists.

Reflections on outcomes: motivation and engagement in game-based training will be achieved if the patient believes in potential success during game play. This perception strengthens the patient's confidence and can be an incentive for him/her to exert more effort to attain the intended game goal. This can be reflected through outcomes that reflect his/her performance in game experience. Moreover, outcomes enable therapists to capture changes in patients' skills, what they are able to do, their level of task performance, and affective reactions. Therefore, reflection on outcome guides therapists to continuously adjust and modify the game according to the patient's existing skills and expectations through therapist-oriented tailoring tools offered by the game environment. To demonstrate the implementation of the proposed framework, a rehabilitation gaming system (RGS) prototype has been developed and described in the next sections.

## 3. Prototype Development

### 3.1. Design Considerations

There are pressing needs for new strategies to increase capacity while optimizing the quality of rehabilitation care. Therefore, the proposed framework for designing game-based intervention for brain injuries cognitive rehabilitation was implemented according to the following design strategy. The first consideration for the prototype development process was to use the web. Development of a web-based rehabilitation platform is a very promising foreseeable solution. Web-based platform can be used in internal networks as well as long-distance public networks for remote patient access from home at no cost. Web is a cross-platform environment that can be accessed from both desktop and mobile platforms regardless of the operating system used. Such systems could be utilized in the clinical setting for inpatient and outpatient rehabilitation. The second consideration for the development process was to use Adobe Flash. This software is a multimedia platform that is well known for creating simulation and games that can be easily viewed on the web, and this is considered as one of the positive factors for using this software in the development process. A negative aspect of using Adobe Flash is the complexity of using the Action Script programming language to create the game system; this was one of the major challenges in this study, which resulted in the time-consuming development process.

### 3.2. Prototype Overview

**Figure 2:**
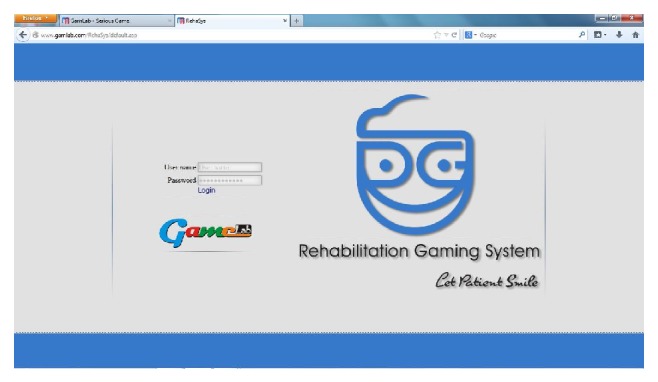
Rehabilitation gaming system main interface.

The main interface of the RGS is shown in Figure 2. The user (i.e., therapist and/or patient) can get into the next level only after filling in the user's name and password fields. In the next sections, therapist modules are described followed by patient's game interfaces.

#### 3.2.1. Therapist

**Figure 3:**
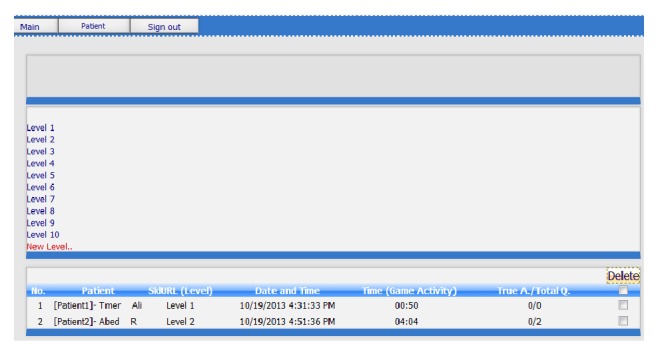
Screenshot of therapist's main interface.

Once the therapist logs into the RGS system, the therapist's main interface will appear as shown in Figure 3. This interface can be described as follows: at the top of the screen there are three buttons (“main,” “patient,” and “sign out”). The “main button” allows therapist to go back to the main interface. The “patient button” allows therapist to add new patient and access and modify patients' information (details in Section (a)). Clicking on the button marked “sign out” exits the user from RGS.

The middle of the screen shows the list of game's levels that were previously created by the therapist. Therapists can edit and modify them by clicking on the game's level name or by clicking on “new level button” to create a new game level (details in Section (b)). The bottom of the screen displays a patient's results. Once the patient finishes the game exercises, therapists can log into the system and see the details such as patient's name, the levels that were played by the patient, the time and date when the patient accessed the game system, and the total amount of time taken by the patient to finish the game level, and the total correct answers are also presented. Therefore, therapists can easily track patient's performance online, which enables him/her to adjust and modify the game's tasks.

**Figure 4:**
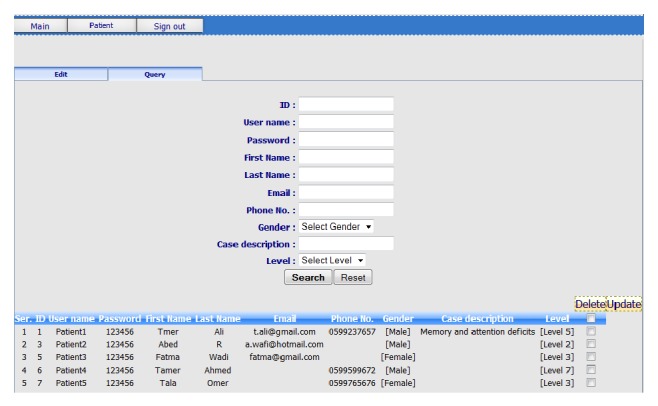
Screenshot of RGS patient editor.

*(a) RGS Patient Editor. *As shown in Figure 4, the RGS patient editor allows therapists to add new patient's information such as user name, password, first name, last name, email, phone number, gender, and case description.

On top of that, RGS patient editor allows therapist to assign game levels to the patients. Also, it allows therapist to search for certain patients through selecting one option/criteria and clicking on the button marked “search.” The system will then generate a query, which would consult the RGS database for the desired information. A list of patients' details will be presented, so therapists can just click on the “check box” that appears beside each patient's record, allowing the therapist to “delete” or “update” patient's information. In the context of this research, at the end, the RGS will be integrated with the management information system of the rehabilitation hospital. Therefore, patient's information will be captured from hospital information system. However, this module (i.e., create and modify patient profile) was purposely developed so that RGS could be used as a standalone system. Moreover, the generated game's interfaces for the patient are textless; also therapists have the ability to use any language according to their patients; hence the RGS can be used worldwide. Furthermore, it offers a new feasible and cost-effective alternative for rehabilitation. 

**Figure 5:**
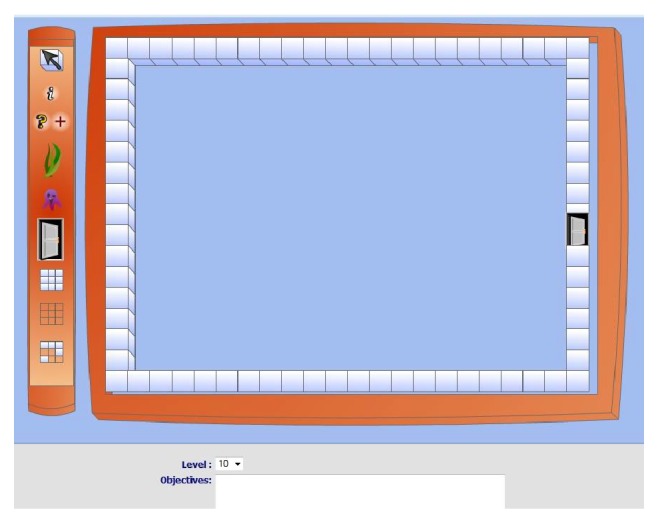
Screenshot of game's design environment.

*(b) Game Design Editor.* There is no limit to the number of mazes that therapists can create. As shown in Figure 5, the game design environment lets therapists create the game and save it. To the right is an empty field surrounded by a border. On the left, there are tools that can be used by therapists to build and tailor the game within the field on the right. Therapists can simply click on a particular tool to activate it and then start the game design process on the empty field. In case of error, like putting the wrong game object in the wrong place, the therapist can simply select the correct one from the “tools” panel and drop it over the wrong one. The game object will instantly turn into the correct choice. At the bottom part of the game design environment, there is a text box and a save button. Therapists can enter texts in the text box describing the objectives of the game and the instructions on how to play the game. When patients log into RGS to play the assigned game, these texts will be launched as a game introductory. In the end, after the therapist completes the design, he/she can simply click on the button marked “save” and the final result will be saved. 

**Figure 6:**
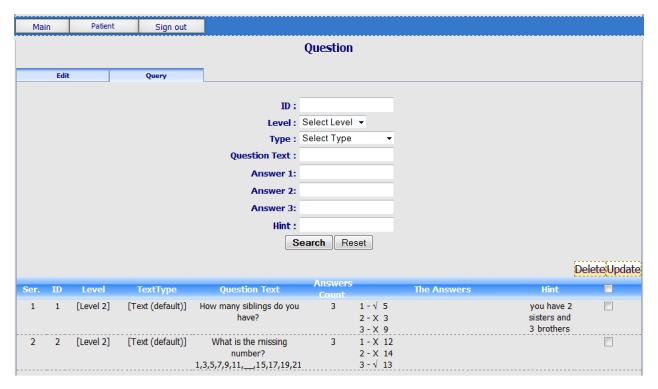
Screenshot of RGS questions editor.

*(c) RGS Questions Editor.* As shown in Figure 6, the RGS questions editor enables therapists to create customized questions with different difficulty levels. Patients will have to answer a number of questions. The questions can be about patient's background such as “How many siblings do you have?” and/or general knowledge questions such as “How many days are there in a week?” and/or mathematical equations such as “5 − 2 = ?” Each question has three possible answers. By clicking on one choice of answers, the game will provide feedback to the patient about his answer. The complexity and the number of questions for patients depend upon the game level designed by the therapist. Patients' answers will allow therapists to analyze the memory and/or cognitive progressions of the patient. 

**Figure 7:**
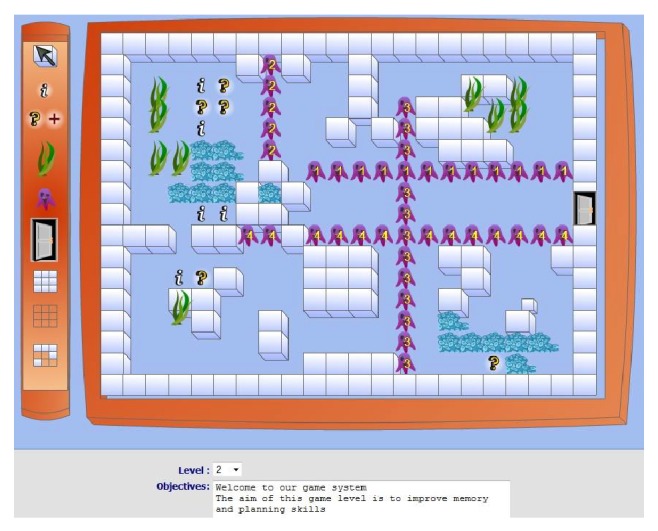
Screenshot of game level design by therapist.

*(d) Creating a New Game Level. *With patient assessment, it is possible to “prescribe” training that targets specific cognitive functions. Therapists, based on their patients' abilities, limitations, and preferences, create this game training. As shown in Figure 7, therapists can access the RGS design environment and start the design process by using the tailoring tools, drawing the maze's pathway, adding the game's objects, identifying the behavior of the opponents, such as how they move or react during game play, and editing and adding questions to the playing field.

#### 3.2.2. Patient Game Interfaces

**Figure 8:**
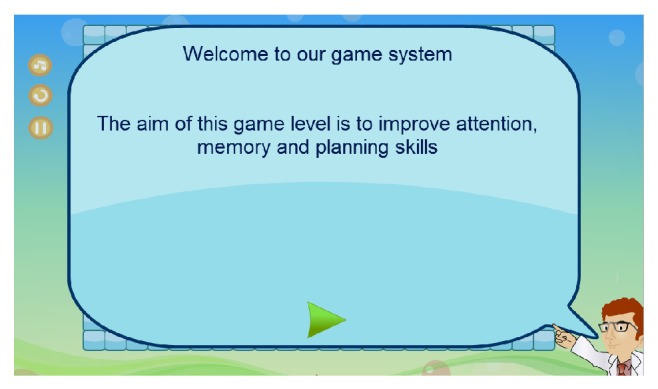
Screenshot of introductory screen.

**Figure 9:**
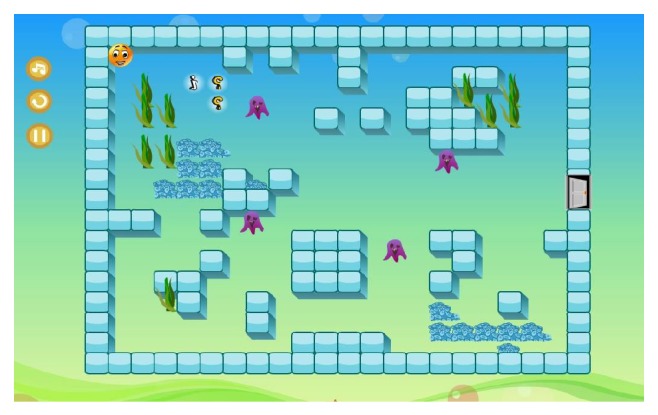
Screenshot of patient's gameplay interface.

Once a patient's profile is created and the game training is tailored and assigned by the therapist, the patient will then have access to a personalized set of game tasks. Patients will have to log into the system. This can be made by either patients or caregivers for those who have interaction difficulties. A short introductory screen first will appear to explain the game's purpose and/or how it is played as shown in Figure 8. Patients can then start the game, as shown in Figure 9, by clicking on the “green arrow button” at the bottom of the screen.

Patients control the yellow smiley face character using the keyboard's arrow keys and steer it from the upper left corner of the playing field to the destination of a maze (i.e., the opened door). Unfortunately, there are opponents blocking the way which are continuously moving. Players need to avoid collision with them to reach the destination. These restrictions force patients to watch out while walking and they must plan their pathway before moving to the maze's destination. Whenever the smiley face hits one opponent while moving, it will fly and then automatically move back to the playing field. The length of time taken to reach the destination is recorded. The quicker the player can reach the destination, the better the results he/she can achieve.

**Figure 10:**
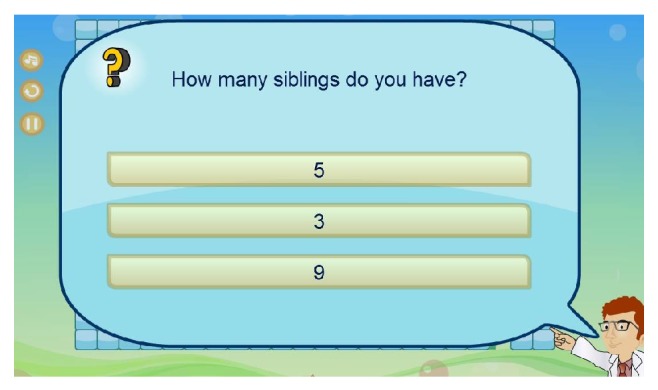
Screenshot of game's question interface.

**Figure 11:**
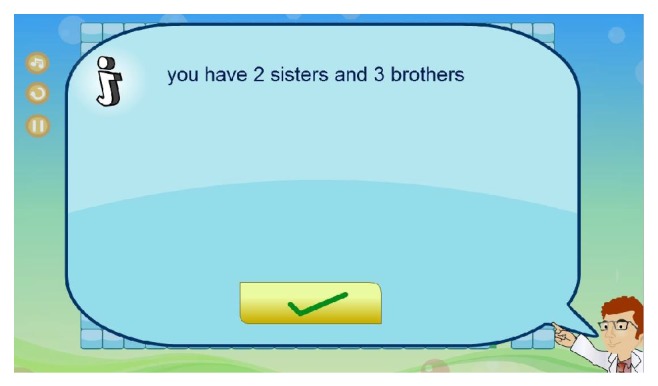
Screenshot of helping aids interface.

Moreover, while patients go through the maze toward the destination, he/she will be asked to answer some customized questions. There are a number of question marks on the playing field. Once the smiley face hits a question mark, it will disappear and the question will be presented via a pop-up screen as shown in Figure 10. The patient will have three choices of answers: when he/she thinks that he/she has found the answer, he/she can click on it and he/she will be given a feedback telling him/her if he/she has chosen correctly. Then, the patient can press the space bar or simply click the “arrow button” to continue playing. In addition, help icons labeled “*i*” are available on the playing field; patients can use them to answer the questions as shown in Figure 11.

Outside the maze, in the upper left corner, three buttons are provided for the patient. The first one allows patients to replay the game level. The second button can pause the game and the last button can stop the game's music. Patients continue until they reach the maze destination (i.e., when the smiley face touches the opened door). The score will be calculated based upon the total time taken to finish the game level and the number of questions the patient has answered correctly.

**Figure 12:**
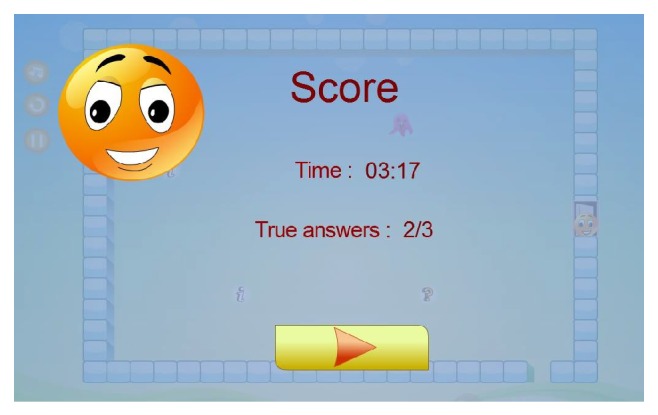
Screenshot of patient's score interface.

The result will be presented as shown in Figure 12 and automatically saved. The patient then will be given the opportunity to go to the next game level by clicking on the “arrow button.”

## 4. Conclusion

In this paper, a conceptual framework for designing game-based cognitive rehabilitation system is presented. This framework can be a useful guide for game designers, developers, and practitioners in designing a rehabilitation gaming system that can significantly affect patients, therapists, and health care systems. Every component of this framework plays a crucial role to provide game-based intervention that can increase rehabilitation effectiveness and competence. To demonstrate the implementation of the framework, a rehabilitation gaming system (RGS) is developed. RGS benefits rehabilitation process and fulfills its real needs such as increasing patient's motivation by providing individualized rehabilitation game experience while simultaneously reducing the development costs associated with it and allowing therapists to track patient's activities and to assess their progress. Furthermore, it is likely to open new opportunities for home-based and unsupervised rehabilitation. The question arises on whether such interventions will be accepted by the target group (i.e., patient and therapist). In the near future, evaluation of the developed rehabilitation gaming system targeting therapists will be conducted to determine their satisfaction.

## References

[1] LoPresti E., Bodine C., Lewis C. (2008). Assistive technology for cognition. *IEEE Engineering in Medicine and Biology Magazine*.

[2] Carmien S., DePaula R., Gorman A., Kintsch A. Increasing workplace independence for people with cognitive disabilities by leveraging distributed cognition among caregivers and clients.

[3] Koenig S. (2012). *Individualized virtual reality rehabilitation after brain injuries [Ph.D. thesis in Human Interface Technology]*.

[4] Tagliaferri F., Compagnone C., Korsic M., Servadei F., Kraus J. (2006). A systematic review of brain injury epidemiology in Europe. *Acta Neurochirurgica*.

[5] Panic A. S. (2010). *Addressing patient motivation in virtual reality based neurocognitive rehabilitation [M.S. thesis]*.

[6] Doidge N. (2007). *The Brain That Changes Itself: Stories of Personal Triumph from the Frontiers of Brain Science*.

[7] Burdea G. C. (2003). Virtual Rehabilitation: benefits and Challenges. *Methods of Information in Medicine*.

[8] Maclean N., Pound P., Wolfe C., Rudd A. (2002). The concept of patient motivation: a qualitative of stroke professionals' attitudes. *Stroke*.

[9] Alankus G., Lazar A., May M., Kelleher C. Towards customizable games for stroke rehabilitation.

[10] Burke W., McNeill M., Charles D., Morrow P., Crosbie J., McDonough S. Designing engaging, playable games for rehabilitation.

[11] Torrente J., del Blanco Á., Moreno-Ger P., Fernández-Manjón B. (2012). Designing serious games for adult students with cognitive disabilities. *Neural Information Processing*.

[12] Elaklouk A., Mat Zin N. Games for cognitive rehabilitation.

[13] Elaklouk A., Mat Zin N. (2012). Requirements for game-based cognitive intervention system for acquired brain Injury. *The GSTF Journal on Computing (JoC)*.

[14] Elaklouk A., Mat Zin N., Shapii A., Zaman H., Robinson P., Olivier P., Shih T., Velastin S. (2013). Game design for acquired brain injury cognitive rehabilitation: a conceptual framework. *Advances in Visual Informatics*.

[15] Scherer M. J., Glueckauf R. (2005). Assessing the benefits of assistive technologies for activities and participation. *Rehabilitation Psychology*.

[16] Tsaousides T., Gordon W. A. (2009). Cognitive rehabilitation following traumatic brain injury: assessment to treatment. *Mount Sinai Journal of Medicine*.

[17] Karlsson P. ICF: a guide to assistive technology decision-making.

[18] Rosenbaum P., Stewart D. (2004). The World Health Organization International Classification of Functioning, Disability, and Health: a Model to Guide Clinical Thinking, Practice and Research in the Field of Cerebral Palsy. *Seminars in Pediatric Neurology*.

[19] Belchior P. D. C. (2007). *Cognitive training with video games to improve driving skills and driving safety among older adults [dissertation]*.

[20] Garris R., Ahlers R., Driskell J. E. (2002). Games, motivation, and learning: a research and practice model. *Simulation and Gaming*.

[21] Perry J. C., Andureu J., Cavallaro F. I., Veneman J., Carmien S., Keller T. (2010). Effective game use in neurorehabilitation: user-centered perspectives. *Handbook of Research on Improving Learning and Motivation through Educational Games*.

[22] Tam S. (2010). *An environment for stroke therapy game authoring [M.S. thesis]*.

[23] Mattheiss E., Kickmeier-Rust M., Steiner C., Albert D. Motivation in game-based learning: it’s more than ‘flow’.

